# 

**Published:** 1881-02

**Authors:** 


### HYMENEAL.

"Domestic happiness, thou only bliss
Of Paradise that has survived the fall."

THOMAS—HARRIS.—In this city, at Christ's Church, John B. Thomas, Jr. and Effie P. Harris. Ceremony by Alex. Waddell, D. D., of Richmond, January 26, 1881, 6 o'clock P. M. The groom is one of our most popular young druggists, and the bride, endowed with beauty of character and person, is the eldest daughter of Prof. Jas. H. Harris, D. D. S., M. D., of Baltimore. We congratulate our young friends and wish them a happy and prosperous future.

MORGAN—EVANS.—Henry W. Morgan, M. D., D. D. S., and Miss Tillie Evans, of Nashville, Tenn. Ceremony at Christ's Church, Nov. 3d, 1880, by Rector B. Graham

WILL *YOU* PLEASE SEND IN *YOUR* SUBSCRIPTION MONEY *NOW?*

*We take the privilege of deciding what we shall publish, but the author only is responsible for his production.*

AFTER MARCH 31ST

THIS JOURNAL

WILL BE ENLARGED,

AND THE

PRICE RAISED

TO

$3 FOR 1881.

Read "POPULAR SCIENCE," P. 83.

This column will be sold for advertising space six month during the year at *special rates.*

Money is at OUR RISK ONLY when sent by Post Office Money Order, Registered Letter, or Check on Banks in New York City or Baltimore, made payable to Dr. B. M. Wilkerson, 68 N. Charles street, Baltimore, Md.

If you prefer to send check on your private bank, unless located in the cities of Baltimore or New York, please add 25 cents for collection.

